# Host–pathogen interaction after infection of *Galleria mellonella* with the filamentous fungus *Beauveria bassiana*

**DOI:** 10.1111/1744-7917.12706

**Published:** 2019-07-22

**Authors:** Lidiia Vertyporokh, Monika Hułas‐Stasiak, Iwona Wojda

**Affiliations:** ^1^ Faculty of Biology and Biotechnology, Department of Immunobiology, Institute of Biology and Biochemistry Maria Curie‐Sklodowska University Akademicka 19 Lublin Poland; ^2^ Faculty of Biology and Biotechnology, Department of Comparative Anatomy and Anthropology, Institute of Biology and Biochemistry Maria Curie‐Sklodowska University Akademicka 19 Lublin Poland

**Keywords:** *Beauveria bassiana*, greater wax moth, host–pathogen interaction, insect body colonization, insect immune response, white muscardine

## Abstract

The filamentous fungus *Beauveria bassiana* is a natural pathogen of the greater wax moth *Galleria mellonella*. Infection with this fungus triggered systemic immune response in *G. mellonella*; nevertheless, the infection was lethal if spores entered the insect hemocel. We observed melanin deposition in the insect cuticle and walls of air bags, while the invading fungus interrupted tissue continuity. We have shown colonization of muscles, air bags, and finally colonization and complete destruction of the fat body—the main organ responsible for the synthesis of defense molecules in response to infection. This destruction was probably not caused by simple fungal growth, because the fat body was not destroyed during colonization with a human opportunistic pathogen *Candida albicans*. This may mean that the infecting fungus is able to destroy actively the insect's fat body as part of its virulence mechanism. Finally, we were unable to reduce the extremely high virulence of *B. bassiana* against *G. mellonella* by priming of larvae with thermally inactivated fungal spores.

## Introduction

Insects and their pathogens constantly undergo antagonistic coevolution. While hosts develop their defense mechanisms, pathogens improve their virulence factors, both to avoid extinction (Joop & Vilcinskas, 2016). Insects have developed a very efficient immune system to deal with numerous infections. They are protected by cuticle, which creates a physical barrier against potential intruders. The trachea as well as the front and hindgut are covered with chitin. Moreover, the conditions in the midgut (low pH and presence of digestive enzymes) do not favour the growth of microorganisms (Wojda, 2017). When the physical and physiological barriers are broken, the presence of intruding microorganisms induces systemic defense response (Moussian, 2010).

*Galleria mellonella* is one of the main insect models used to study innate immunity and host–pathogen interaction and to test antimicrobial drugs *in vivo*. Its larvae live in beehives or, more often, in stored honeycombs. They feed on wax, honey, and pollen (Wojda, 2017) causing a disease called *galleriosis*. When microorganisms enter the hemocel, for example *via* injured cuticle or through an injured gut, *Galleria mellonella* hemocytes (plasmatocytes and granulocytes) immediately engulf them. Moreover, clusters of microorganisms can be enclosed in capsules that are assembled from the hemocytes (Tojo *et al*., 2000; Lavine & Strand, 2002). Infection can also activate signalling pathways that lead to the activation of NF‐κB‐like transcription factors—Dif and/or Relish, which regulate the expression of genes encoding antimicrobial peptides. As a result, antimicrobial peptides appear in the insect hemocel (Mak *et al*., 2010). They mainly perforate microbial membranes, which results in absence of homeostasis in the microorganism and, therefore, its death (Cytryńska, 2009). Besides antimicrobial peptides, *G. mellonella* possesses some polypeptides that are permanently present in larval hemolymph and whose amount can change after infection. These include lysozyme, that is, a muramidase digesting bacterial peptidoglycan, and apolipophorin III, which is a so‐called storage protein responsible for lipid transport to muscles during flight, but also enhancing immune properties of hemolymph (Brown *et al*., 2009; Mak *et al*., 2010).

Natural pathogens of *G. mellonella*, which have coevolved with this moth, have developed mechanisms enabling to force defense barriers of the host and establish a biotope inside the larval body where they can proliferate. Many of these pathogens, such as *Bacillus thuringiensis* and *Beauveria bassiana*, are not toxic to humans and are used as bioinsecticides (Nielsen‐LeRoux *et al*., 2012; Lacey *et al*., 2015). *Beauveria bassiana* is a fungus with a dual life cycle: as a plant endophyte and as an entomopathogen (Barelli *et al*., 2016). When fungal propagules meet insect cuticle, they attach to it and form structures allowing invasion. The high turgor of the penetration peg provided by high glycerol content allows the fungus to force a way into the insect body. An intruding pathogen secretes enzymes such as chitinases, lipases, and proteases that digest host's tissues, which in turn serve as a rich source of nutrition for the invading fungus (Mondal *et al*., 2016). After reaching the hemolymph, *Beauveria bassiana* grows as single yeast‐like cells with a very thin cell wall called blastospores. The shift to blastospores allows reduction of the number of pathogen‐associated molecular patterns (PAMPs), that is, to minimize recognition and therefore the defense response. *Beauveria* proliferates in the insect body until depletion of nutrients (Pendland *et al*., 1993; Tartar *et al*., 2005). Then, the fungus induces hyphal growth and penetrates the cuticle, this time outside. Finally, the sporulating fungus covers the entire insect body and is able to infect another host. The insect disease caused by *Beauveria* is called *white muscardine* (Mascarin & Jaroński, 2016).

Besides blastospores, *B. bassiana* produces submerged conidia and aerospores. The latter are formed on solid medium, while blastospores and submerged conidia emerge on rich and poor liquid medium, respectively. These three types of cells differ in their physical properties, but all of them are infectious (Holder & Keyhani, 2005; Holder *et al*., 2007). During infection, *Beauveria* secretes virulence factors, including insecticidal depsipeptides beauvericin and bassianolide, cyclosporin A, which inhibits the phagocytic activity of *Galleria* plasmatocytes, insecticidal oosporein, and others. *B. bassiana* kills the infected host by mechanical destruction of the host tissues with the growing mycelium and by the action of secreted secondary metabolites (Vilcinskas *et al*., 1999; Fang *et al*., 2005; Gibson *et al*., 2014). Recently, genes involved in *B. bassiana* infection of *G. mellonella* have been identified (Fan *et al*., 2017; Chen *et al*., 2018). The set of genes whose expression level changes upon the infectious process depends on the host (Zhou *et al*., 2018)

In this article, we present the *Beauveria bassiana* infection process in the greater wax moth larvae *Galleria mellonella*. We have analyzed how this natural pathogen colonizes the larval body and how the host develops antifungal response to natural infection and to injection of blastospores.

## Materials and methods

### Insects and infection

*Galleria mellonella* (Lepidoptera: Pyralidae) were reared on honeybee nest debris at 28 °C in darkness. *Beauveria bassiana* strain 80.2 was a kind gift from Dr Bruno Lemaitre (CNRS, CGM, Gif‐sur‐Yvette, France). Natural infection was done by gentle rolling each larva for 30 seconds on the surface of sporulating fungal culture in a Petri dish and leaving larvae on that plate for 6 min (Lemaitre *et al*., 1997). The number of aerospores that attached to the larvae was calculated after washing them out from the cuticle with the use of dichloromethane (Ment *et al*., 2010). The number was estimated on (2.6 ± 0.6) × 10^6^ per larva. *G. mellonella* was also infected by injection of *B. bassiana* blastospores or with an indicated number of *Candida albicans* (ATCC 10231) cells.

*C. albicans* to be injected was cultivated on YPD medium (1% yeast extract, 2% bactopepton, 2% glucose) at 30 °C with 120 r/min shaking. The fungal cells were centrifuged at 5500 × *g* and suspended in phosphate buffered saline (PBS, 140 mmol/L NaCl, 2.68 mmol/L KCl, 10 mmol/L Na_2_HPO_4_, 1.76 mmol/L KH_2_PO_4_, pH 7.4) to the desired density. The number of fungal cells was estimated by counting colony‐forming units grown on the plates after sowing the suspension with known optical density (OD at 600 nm). Before the injection, the surface of the larva was sterilized with 70% ethanol. After infection, the larvae were reared in well‐ventilated plastic boxes supplied with food, at 28 °C.

### Preparation of fungal blastospores

*Beauveria bassiana* was grown on Sabouraud Dextrose Agar (1% polypeptone, 4% glucose, 1.5% agar) supplemented with 2% yeast extract. For preparation of blastospores, the fungus was grown on SL YPD medium (4% glucose, 2% bactopeptone, 0.5% yeast extract) for 3 d at 25 °C with gentle shaking. The culture was then filtered through sterile miracloth and centrifuged as described (Wojda *et al*., 2009). The number of blastospores in the water suspension was counted in a Burker chamber. The spores were kept at −20 °C for further use. For their heat inactivation, the fungal blastospores were diluted in apyrogenic water to the desired density and heated at 60 °C for 15 min. The suspension was then cooled down, mixed, and injected to the larvae in the volume of 5 *µ*L as described above. To check whether the heat‐treated blastospores were able to germinate, they were spread on a plate with Sabouraud Dextrose Agar supplemented with yeast extract. No fungal colony appeared.

### Preparation of cell free hemolymph and testing its defense activity

The larvae were anesthetized in ice‐cold water, sterilized with 70% ethanol, and injured with a sterile needle. Hemolymph was collected to Eppendorf tubes kept on ice and containing a few crystals of phenylthiourea to prevent hemolymph melanization. Hemolymph pooled from ten larvae in each group was centrifuged at 200 × *g* for 5 min and then at 10 000 × *g* for 10 min, both times at 4 °C. The cell‐free hemolymph was divided into portions to avoid repeated freezing and unfreezing and kept at −20 °C until use. Antifungal activity was tested with a diffusion assay on Potato Dextrose Agar medium (PDA, 50 g grated filtered potato/1000 mL, 0.5% glucose) with 0.7% agar, containing spores of *Aspergillus niger* (strain 71.1 obtained from the collection of the Department of Genetics and Microbiology, Maria Curie‐Sklodowska University, Lublin). To prepare plates, the fungus was grown on PDA medium containing 1.6% agar at 28 °C. Fungal spores were obtained by flushing PDA slants overgrown with the fungus with sterile water. The spores were separated from the remaining mycelium by filtration through cotton wool and counted in the Burker chamber. Then, spores were added to PDA, when the temperature dropped to ca. 44 °C and 8 mL of medium containing 1.6 × 10^6^ spores was poured into Petri dishes. Hemolymph containing 160 *µ*g of proteins in a volume of 4 *µ*L was applied into 2.7 mm holes made in the PDA. The protein concentration was estimated with the Bradford (1976) method. The plates were then incubated for 24 h at 28 °C. The growth inhibition zones were measured and converted into an amphothericin B equivalent on the basis on the standard curve made with a known concentration of amphotericin B (Sigma‐Aldrich, Germany).

### Histological analysis of infected larvae

Routinely, the *G. mellonella* larval tissues were fixed in phosphate‐buffered 4% paraformaldehyde for 7 d, dehydrated, and embedded in Paraplast (Sigma‐Aldrich, St. Louis, Mo, USA). Next, they were sectioned with a rotating microtome to obtain 5 *µ*m thick paraplast sections, which were mounted on polysine‐coated glass slides (Thermo Fisher Scientific, Braunschweig, Germany). Next, the larval sections were deparaffinized in xylene (2 times for 5 min), rehydrated by passing through decreasing concentrations of alcohols (100%, 95%, and 70% for 5, 3, and 3 min, respectively), and stained with hematoxylin (Sigma‐Aldrich) for 7 min. The sections were then rinsed for 20 min with running tap water and deionized water for 5 min. After that step, the sections were stained with eosin (15 min, Sigma‐Aldrich) and rinsed again. Next, the slides were dehydrated for 3 min in each alcohol solution (70%, 95%, 100%) and embedded in xylene dibutyl phthalate (DPX, Sigma‐Aldrich). Two solutions were used for tissue staining with calcofluor white: solution A (containing 9% KOH and 9% glycerol) and solution B (containing 0.1% calcofluor white, Fluka, Switzerland). Immediately before staining, solutions A and B were mixed in a 1: 1 ratio and the larval sections were immersed in the mixture for 10 min in the dark. All specimens were analyzed using a Carl Zeiss Axiovert 200M confocal microscope (Germany).

### Counting hemocytes in the hemolymph of G. mellonella larvae

At the indicated time‐points after natural infection, the larvae were cooled down on ice and bleed and the hemolymph was loaded directly in the Burker chamber. The Olympus BH‐2 phase‐contrast microscope was used to count hemocytes. The number of each type of hemocytes per unit of hemolymph volume was calculated in a standard way designated for the Burker chamber and then re‐calculated into percentage in relation to all hemocytes.

### Statistics

Where indicated, One Way ANOVA with Tukey's *post hoc* test and Student's *t*‐test were used. Significance was established at *P* < 0.05. Normality of data was checked with the Shapiro–Wilk test. Survival analysis was done with the use of a log‐rank test (Goel *et al*., 2010).

## Results

### Body colonization by the infecting fungus

**Figure 1:**
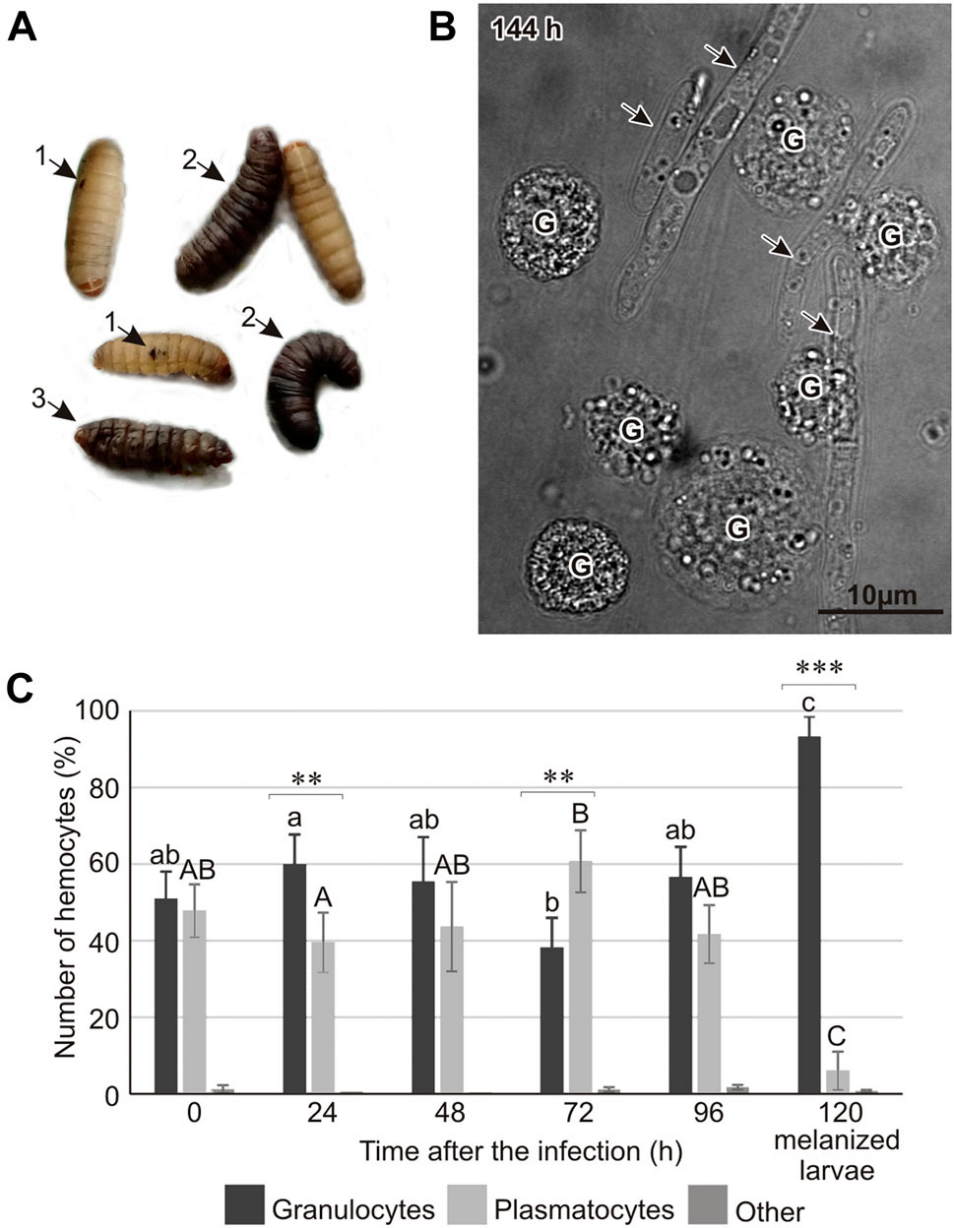
(A) *G. mellonella* larvae naturally infected with *Beauveria bassiana*. Larvae with punctate (1) and complete (2) melanization of the cuticle as well as dead individuals (3) were observed 120–144 h after the natural infection. (B) Hyphal growth of *B. bassiana* (indicated by arrows) in the hemolymph of *G. mellonella* larvae at the advanced stage of infection (144 h, live larva with a melanized cuticle) viewed under a confocal microscope. G, granulocytes. (C) The number of granulocytes and plasmatocytes in the larval body at the indicated time points after natural infection of *G. mellonella* with *B. bassiana*. Different letters symbolize statistically significant difference between the percentage of a given type of hemocytes (lowercase and capital letters for granulocytes and plasmatocytes, respectively, One way ANOVA, *post hoc* Tukey's test, *P* < 0.05). Significant differences between the number of plasmatocytes and granulocytes within particular time‐points are indicated by asterisks (**P < 0.01; ***P < 0.001; Student's *t*‐test).

We have previously published the survival curve for *G. mellonella* larvae infected naturally with *B. bassiana*: 70% of infected larvae lived 144 h after attachment of conidia to the cuticle, while 50% survived at 160 h. After 192 h, only 10% survived, and all infected larvae were dead after 216 h (Wojda *et al*., 2009). The rate of the infection process differed between individuals. After 120–144 h, we observed dead larvae and larvae with punctate or total melanization of the cuticle (Fig. 1A). The last ones died within 24 h. In hemolymph of individuals with punctate melanization, we observed single cells of *B. bassiana* (data not shown), while numerous, not surrounded by hemocytes, fungal hyphae were visible in the hemolymph of individuals with total cuticle melanization (Fig. 1B). Additionally, at the advanced stage of infection, granulocytes were the most numerous types of hemocytes in the hemolymph of the totally melanized larvae (Fig. 1C).

**Figure 2:**
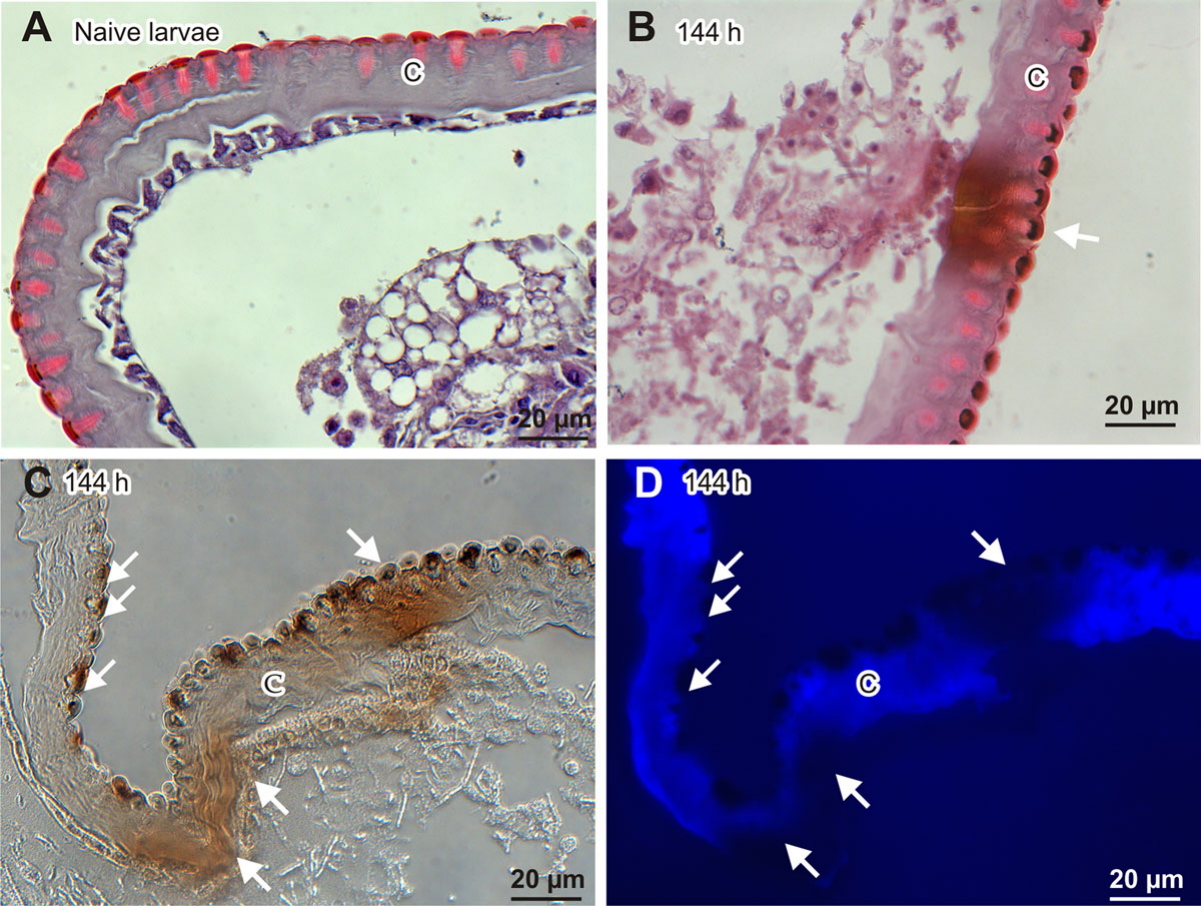
Cuticle of a healthy larva (A) and larvae in the advanced stage of *B. bassiana* infection (B–D; 144 h after the natural infection). (A, B) Hematoxylin/eosine staining; (C) no staining, viewed under visible light; (D) staining with calcofluor‐white, which binds to chitin, viewed under UV light. The cuticle is melanized deeper in sites where local loss of chitin was detected. C, cuticle; the arrows indicate melanization sites.

**Figure 3:**
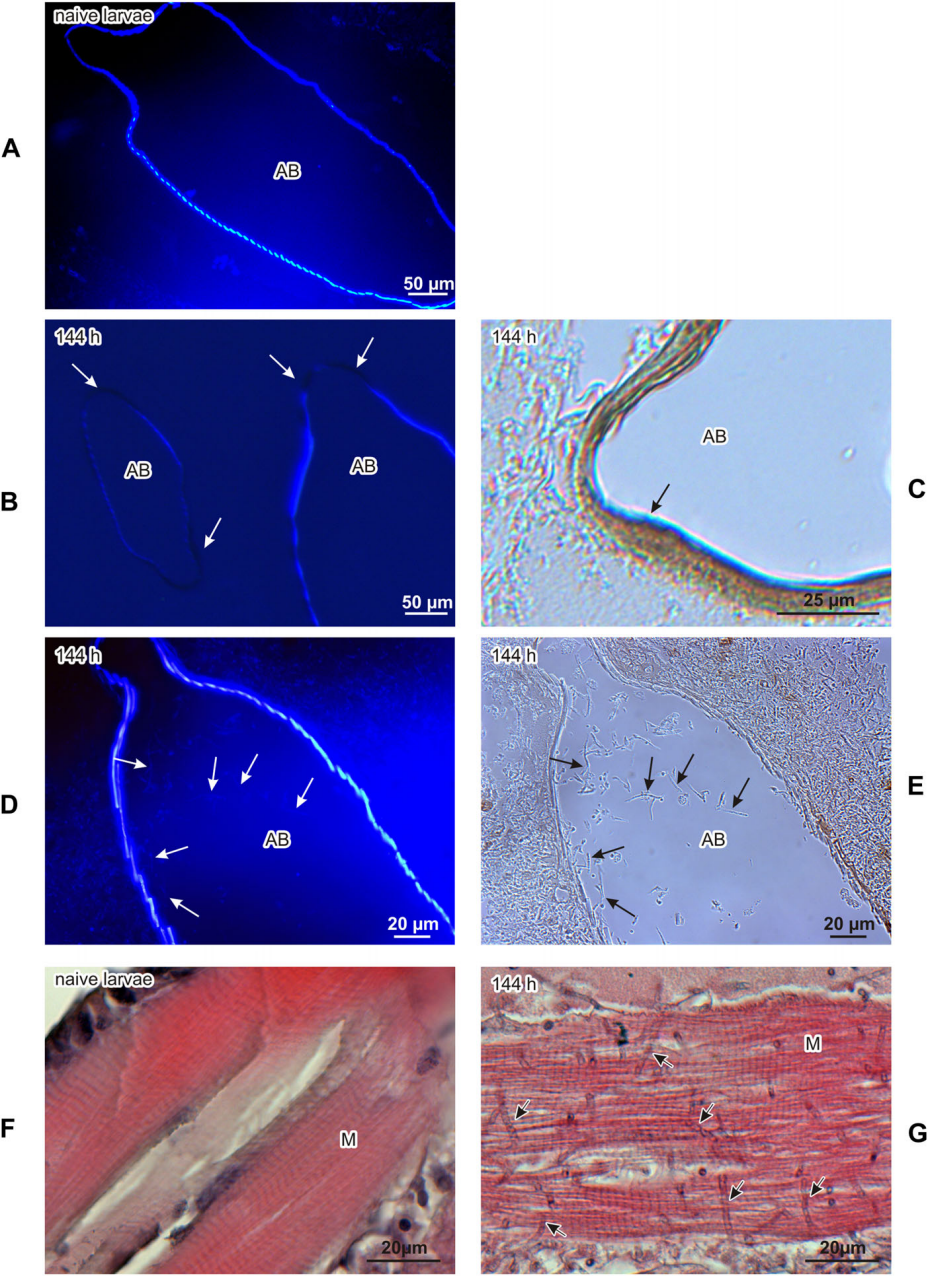
Air bags of healthy (A) and infected (B–E) *G. mellonella* larvae. (A, B, D) Staining with calcofluor‐white, observed under UV light; (C, E) no staining, seen under visible light. (A) Air bag of a healthy larva. (B) Disturbed walls of air bags have no ability to bind calcofluor‐white (indicated by arrows). (C) Melanized wall of air bag is indicated. (D) Calcofluor‐white binds to chitin lining the walls of the air bags and to chitin present in the thin cell walls of *B. bassiana* (indicated by arrows) growing inside the air bags. (E) Hyphae of *B. bassiana* (indicated by arrows) seen under visible light. Muscles of healthy (F) and infected (G) *G. mellonella* larvae (144 h after natural infection). A growing fungus is indicated by arrows. AB, air bags; M, muscles.

**Figure 4:**
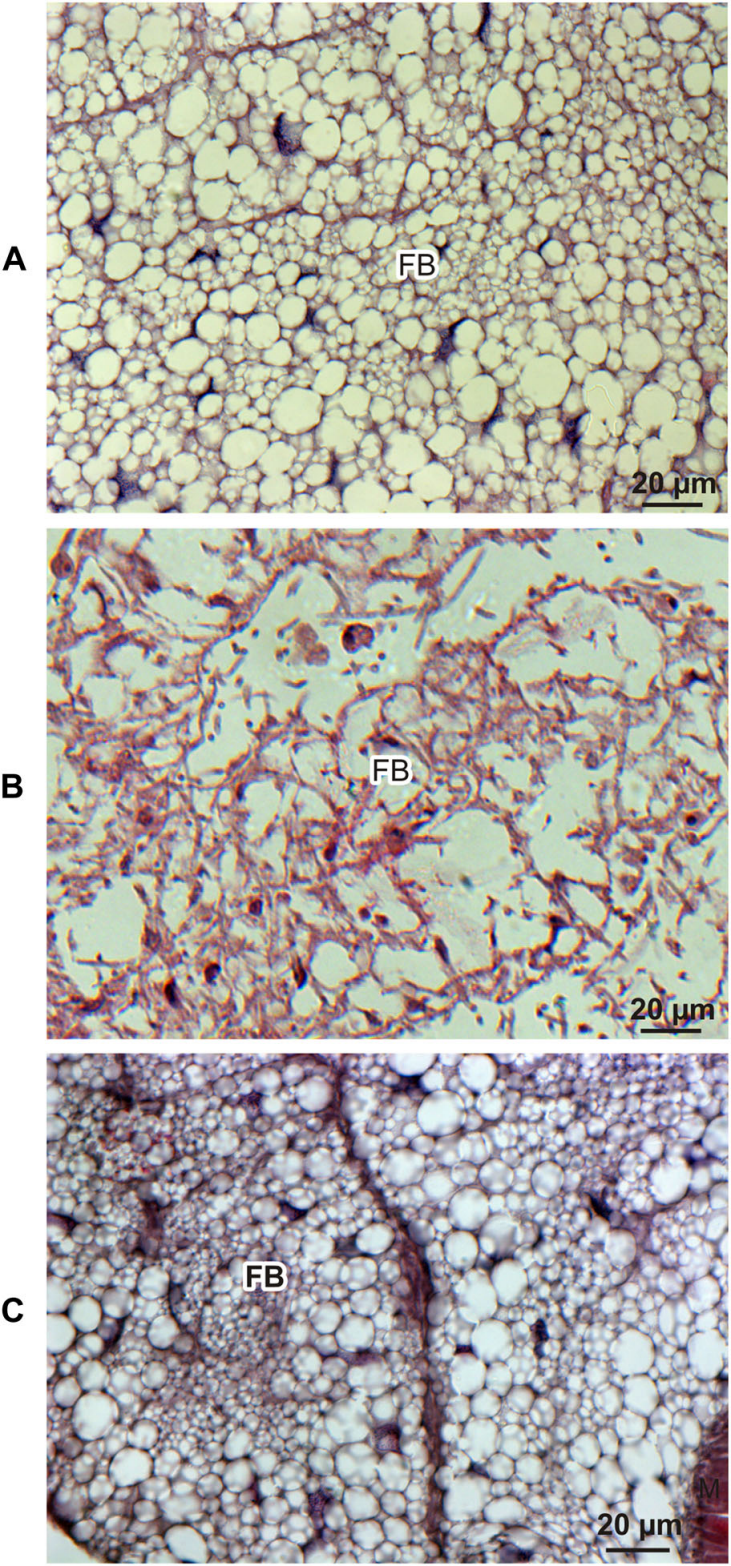
The fat body of a naive (A) *G. mellonella* larva, a larva at the advanced stage of infection with *B. bassiana* (144 h after the natural infection; B), and a larva after injection of the lethal dose of *C. albicans* (72 h after intrahemocelic injection of 2 × 10^5^ cells of *C. albicans*; C).

Further histological analysis showed that the melanization of cuticle was accompanied by an inability to bind calcofluor, which indicates local chitin losses (Fig. 2). Besides the cuticle, we also observed melanization of some air bags and a local inability to bind calcofluor white (Figs. 3B, C). Additionally, some air bags were populated by the infecting fungus (Figs. 3D, E). The analysis of muscles revealed the presence of the fungus, which may have been one of the causes of the lower motility of the infected larvae (Fig. 3G). The most spectacular effect of the fungal colonization was observed in the fat body, that is, the main organ responsible for the synthesis and secretion of defense molecules into hemolymph. This organ lost its spongy structure and was transformed into an irregular formation that was completely overgrown by the fungus. To compare, the fat body was not destroyed at the advanced stage of infection caused by *Candida albicans* (Fig. 4).

### Defense response and survival of G. mellonella infected with B. bassiana

**Figure 5:**
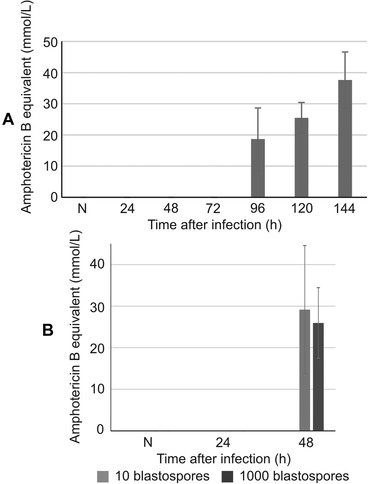
Antifungal activity of the hemolymph of *G. mellonella* larvae naturally infected with *B. bassiana* (A) and after injection of 10^1^ and 10^3^ blastospores (±SD; B) at the indicated time‐points after infection; N denotes naive larvae (no activity detected). The experiment was performed in three biological replicates.

The insect body was occupied by *B. bassiana* despite the appearance of antifungal activity in the hemolymph of the naturally infected larvae. This activity was detected 96 h after natural infection and persisted to some extent at the further time‐points (Fig. 5A). Since natural infection does not allow studying the dependence between insect immune response and the dose of the infecting fungus, we infected the larvae by direct injection of blastospores into the hemocel. With this method, the first stage of infection, that is, fungal adhesion and cuticle penetration, is omitted. However, it allows monitoring the stages of infection from the moment when the fungus reaches the hemocel and multiplies in the form of blastospores. We assayed the defense activity in the hemolymph of larvae injected with two doses of blastospores: 10^1^ and 10^3^. Antifungal activity appeared 48 h after the injection and was similar for both doses. The level of this activity was also similar to that of naturally infected insects (up to 50 mmol/L equivalent of amphotericin B). It was impossible to compare the activity 72 h after the infection with two doses of blastospores due to the moribund stage of larvae injected with 10^3^ cells. However, 72 h after the infection with 10 blastospores, the antifungal activity increased to an equivalent of 200 mmol/L amphotericin B (not shown in the figure), indicating that the injected spores induced a higher level of defense reaction than in the case of the natural step‐wise fungal infection.

**Figure 6:**
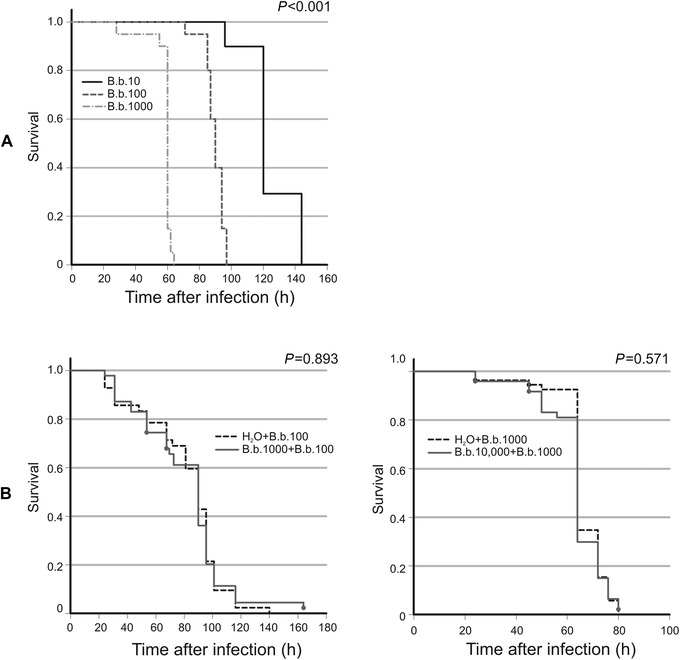
(A) Survival rate of *G. mellonella* larvae injected with the indicated number of *B. bassiana* (*B. b*.) blastospores. The analysis was performed on the total number of 100 individuals in each group. (B) Survival rate of larvae pretreated with the indicated number of heat‐killed *B. bassiana* (*B. b*.) blastospores, injected into the hemocel and then, 48 h later, injected with the indicated number of live *B. bassiana* cells. Pretreatment with heat‐killed blastospores did not increase resistance to subsequent infection in comparison to the control group, which received apyrogenic water (H_2_O) in the first injection. The analysis was performed on the total number of 60 (left) and 50 (right) individuals in each group. Larvae double injured did not show mortality at the indicated time points (data not shown).

The survival time of an infected larva depended on the injected dose of *B. bassiana* spores. The shortest time was noted after the injection of 10^4^ blastospores, when all animals died within ca. 60 h, while the injection of 10^2^ spores resulted in death of all animals within ca. 100 h (Fig. 6A). After injection of ca. 10^1^ blastospores, the insects slowly died within 6 d, as presented before (Vertyporokh & Wojda, 2017). We further checked whether the very high virulence of *B. bassiana* toward *G. mellonella* could be reduced by priming. The larvae were preinjected with thermally inactivated spores and then, 48 h later, with invasive fungal propagules. We did not use the dose of 10^1^ cells because adding 48 h to the survival time (6 d) exceeds the duration of a particular larval stage. The “primed” larvae were injected with 10^2^ and 10^3^ blastospores. The pretreatment with heat‐treated blastospores did not reduce the mortality of *G. mellonella* larvae (Fig. 6B).

## Discussion

The filamentous fungus *Beauveria bassiana* is a natural pathogen of the greater wax moth *Galleria mellonella*. It has developed mechanisms facilitating access to the insect body *via* the cuticle (Boucias *et al*., 2018). Attached to the cuticle, the fungus secretes enzymes: proteases, chitinases, and lipases, which digest insect body's cover and simultaneously exert the mechanical pressure on host's tissues (Butt *et al*., 2016). On the other hand, infected insects produce and secrete defense molecules to the hemolymph, such as antimicrobial peptides acting against the intruder or inhibiting its virulence factors (Vilcinskas & Wedde, 2002; Wedde *et al*., 2007; Vertyporokh & Wojda, 2017; Trevijano & Zaragoza, 2019). Thus, the body of an infected insect is a place of war for death and life between the host and its pathogen. The organism whose mechanisms of defense or virulence will appear to be more effective survives.

We have found that, during *B. bassiana* invasion, melanin is deposited in the cuticle in sites of low calcofluor‐white binding due to the lack of chitin. This probably prevents loss of fluids. The first signs of melanization occur concurrently with the presence of fungal hyphae in insect's body fluids. During the infection process, we detected slight modulation of the proportion between plasmatocytes and granulocytes, which ended up with almost complete disappearance of plasmatocytes in the hemolymph of melanized larvae. Almost all of the visible cells were granulocytes. It is likely that the invading fungus either disturbs plasmatocytes or interferes with their differentiation. Plasmatocytes secrete the so‐called plasmatocyte‐spreading peptide that acts as a cytokine attracting other hemolymph cells to the site of infection for nodule or capsule assembly (Johansson, 1999). The absence of plasmatocytes can make the encapsulation of growing hyphae impossible; hence, the fungus can freely develop in the hemolymph, as shown here. Melanin was deposited also in the walls of air bags. Air bags are extensions of tracheas in which oxygen, whose high amount is necessary for wax digestion, is deposited. Like trachea, air bags are covered by a chitin layer that creates a mechanical barrier against intruders. Probably, the intruder is able to force this barrier with the use of extracellular enzymes. We have found that, inside air bags, the fungus finds air environment where it can grow in the form of hyphae. Ventilation of these structures may promote dispersal of aerospores. In addition to air bags, the intruder colonizes muscles, which may explain the lower motility of infected larvae in comparison to healthy ones. The most spectacular, however, was the complete destruction of the insect fat body by the infecting fungus. This is consistent with the results obtained by Xiong *et al*. (2013), who showed a loss of the cellular structure of the fat body in the peach fruit moth *Carposina sassakii* infected with *B. bassiana*. The ability of entomopathogenic fungi to destroy the insect fat body is probably an effect of antagonist host–pathogen coevolution (Joop & Vilcinskas, 2016). It is known that this organ is the main source of defense molecules secreted to the hemolymph. Its destruction makes the insect host unable to perform its physiological function, including the immune‐induced synthesis of defense molecules. Destruction of the fat body is not a common mechanism of pathogen virulence. We showed that the infection of *G. mellonella* larvae with the human opportunistic pathogen *Candida albicans*, which has not coevolved with *G. mellonella*, did not result in the destruction of this organ, even in the very advanced stage of the infection. It seems that, during coevolution, *B. bassiana* somehow “has learned” that the destruction of the fat body results in the easiest colonization of the host. Indeed, the virulence mechanisms of *Beauveria* are extremely effective, as there is no sublethal dose for blastospores injected to the larval hemocel. Regardless of whether 10^1^ or 10^4^ *B. bassiana* blastospores reach the hemocel, the triggered antifungal response is similar 48 h after injection. We have shown previously that the expression of genes encoding defense peptides was not higher after the injection of 10^3^ blastospores than after 10^1^. Even more, in the case of the higher dose at the late time‐points (72 h), we observed a reduced amount of gallerimycin, galiomycin, and IMPI (inhibitor of metalloproteinases from insects) transcripts in comparison with their amount observed at 48 h (Vertyporokh & Wojda, 2017). At that time, the fat body was not destroyed yet, suggesting a different mechanism of the possible inhibition. The inability of *G. mellonella* to survive the intrahemocelic injection of the low number of fungal blastospores is consistent with our earlier observation (Vertyporokh & Wojda, 2017). We showed that the survival time after the spore injection was strictly correlated with the number of spores: compare 60 h after injection of 10^4^ blastospores and 6 d after injection of 10 cells. This means that when *B. bassiana* spores enter the larval body, insects have no chance to survive. This is consisted with the observation reported by Dubovskiy *et al*. (2013a). The analysis of the host–pathogen micro‐coevolution of *G. mellonella* and *B. bassiana* revealed that the 25th insect generation was more resistant to the fungus. The comparison between selected and nonselected lines showed that the selected (more resistant) line had higher expression of some immune defense genes in the cuticle and epidermis, but not in the fat body. Also the same authors has showed that the melanotic strain of *G. mellonella* with a thicker and more sclerotic cuticle due to the higher melanin content was more resistant to *B. bassiana* (Dubovskiy *et al*., 2013b). All this confirms that the main strategy of *G. mellonella* against *B. bassiana* is to invest in the frontline of defense, since the insect will not survive when the fungus enters the body cavity.

The pretreatment of *G. mellonella* with the thermally inactivated blastospores did not increase the insect ability to survive the injection of blastospores. Moreover, when pretreatment of larvae with low doses of *Bacillus thuringiensis* and *C. albicans* increased the resistance against high doses of the same pathogens, we did not observe higher survival rates in insects injected with *B. bassiana* cells after pretreatment with *B. thuringiensis* and *C. albicans* (Taszłow *et al*., 2017 and data not shown). Such an effect, called immune priming (enhanced resistance to the pathogen after being exposed to the sublethal dose), observed for entomopathogenic bacteria *B. thuringiensis* (Taszłow *et al*., 2017) and fungus *C. albicans* (Bergin *et al*., 2006), is not the case for *B. bassiana*. The difference may be related to the fact that there is no nonlethal dose of *B. bassiana* spores infecting *via* injury. Immune priming also occurs after injection of dead cells, which may trigger immune response due to the presence of pathogen‐associated molecular patterns (PAMPs) (Dunphy *et al*., 2003; Wu *et al*., 2014). However, dead cells do not produce virulence factors, which may also trigger immune response acting as a “danger” signal, or by a negative influence on host's cells as a damage signal (Ming *et al*., 2014). It is likely that virulence factors take part in achieving the immune priming effect. The host–pathogen coevolution can be described with the trench warfare model, according to which defense and virulence alleles giving advantages to a host or pathogen oscillate in populations cyclically (Tellier *et al*., 2010). It is likely that now the entomopathogenic fungus *B. bassiana* has an advantage over its natural host *G. mellonella*, which has no chance to survive if fungal spores reach the interior of its body.

## Disclosure

The authors declare no conflict of interest.
